# Caste- and sex-specific differential investment in brain regions of Australian ants

**DOI:** 10.1016/j.isci.2026.116039

**Published:** 2026-05-23

**Authors:** Saroja Ellendula, Zachary B.V. Sheehan, Marcel E. Sayre, Fleur Ponton, Ajay Narendra

**Affiliations:** 1 School of Natural Sciences, Macquarie University, Sydney, NSW 2109, Australia

**Keywords:** Biological sciences, Zoology, Entomology, Neuroscience

## Abstract

Insect species have distinct lifestyles and behaviors, which require unique information processing requirements. Ants are unusual in that closely related individuals within a single species exhibit distinct lifestyles and locomotory strategies: workers are exclusively pedestrian and engage in foraging and colony maintenance tasks; alate males lead a life exclusively on the wing and are tasked with locating females; alate females exhibit both modes of locomotion and engage in mating, colony establishment and foraging. Here, we aim to identify within a single species the neural adaptations required for carrying out specific information processing tasks. We addressed this question by comparing volumes of functionally distinct brain regions in three castes of two Australian ants*.* We found that female castes had larger brains with pronounced mushroom bodies, supporting their broader behavioral repertoire and navigational tasks. Male ants had enlarged optic lobes and central complexes, highlighting the significance of vision and orientation in mate searching.

## Highlights

• Neural investment between ant castes varies with lifestyle and ecology
• Male ants have the smallest brains, yet the largest optic lobes and central complexes
• Worker ants that rely on visual memories have the largest mushroom body

## Graphical abstract

**Figure:**
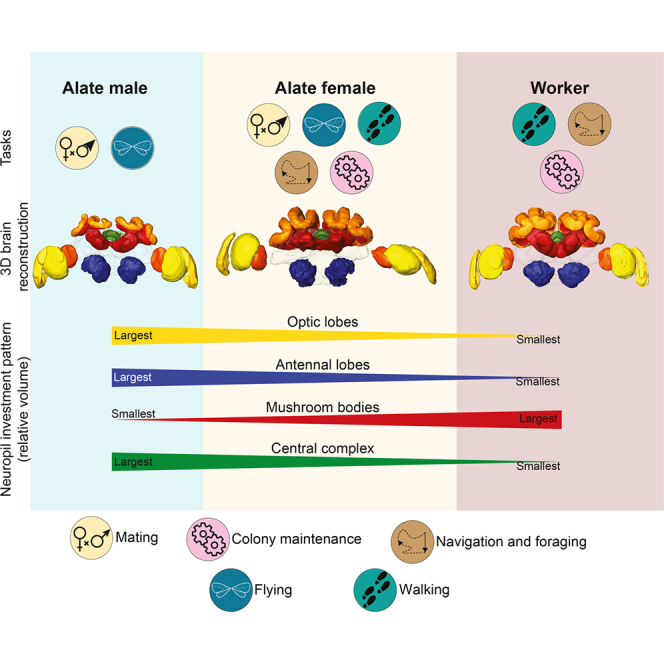

## Introduction

Insects offer an unparalleled opportunity to explore the neural basis of behavior and cognition in an evolutionary context. Though an insect brain weighs a milligram and contains orders of magnitude fewer neurons than a vertebrate brain, these miniaturized brains support sophisticated navigation, communication, decision-making, spatial memory and learning. Compartmentalized organization of insect brains into discrete neuropils, which are functionally specialized processing centers, provides an ideal system to investigate how evolutionary pressure shapes neural investment strategies.^1^^,^^2^

Insect brains exhibit remarkable plasticity, with neuropil volumes, cell numbers and connectivity varying with lifestyle and information content in the environment. One brain region that is often implicated in experience dependent plasticity is the mushroom body, a higher-order center involved in associative learning and memory across insect taxa. Butterflies that exhibit complex foraging behaviors and enhanced long-term memory possess larger mushroom bodies compared to those that exhibit simpler behavioral repertoires.^3^^,^^4^ Similarly, experienced foragers tend to have larger mushroom bodies compared to similarly aged animals that stay within the confines of the nest.^5^^,^^6^^,^^7^^,^^8^ Changes to lifestyle also reshape insect brain architecture. This is evident in the central complex, a highly conserved sensorimotor integration center that encodes internal representations of head direction and goal direction and drives steering commands.^9^^,^^10^^,^^11^ In locusts, the central complex increases markedly in volume during the transition from a solitary to a gregarious phase, an adaptation likely to support movement and coordination within dense swarms.^12^ Insect brains, thus, have the capacity to reorganize neural structure in response to change in lifestyles, sensory demands, and ecological contexts.

Ants provide a unique opportunity to study the link between brain, behavior, and ecology within a species, due to the distinct lifestyles exhibited by the different castes. Workers, the most populous caste, are typically sterile females that are exclusively pedestrian and perform the largest share of tasks within the colony, including brood care, nest maintenance, and foraging. For successful foraging, workers rely on different sensory modalities to locate food sources and to find their way back home.^13^ In contrast, the reproductive castes, alate males and females, have a limited behavioral repertoire focused primarily on mating and colony founding.^14^ Alate males and females rely on visual information for flight control, navigation, and obstacle avoidance. Males also use visual cues to track fast-moving conspecific females and fend off competitors. While males die after mating, alate females shed their wings and establish new nests as queens, spending the rest of their lives in the darkness. In some species (e.g., *Myrmecia*), queens forage until the first brood of workers emerges.^15^^,^^16^

Variation in lifestyle between castes and species is reflected in the organization of their external sensory array. Male ants tend to have disproportionately large compound eyes that contain more and smaller lenses in each eye compared to any other caste.^17^^,^^18^^,^^19^ Among all castes, males possess the largest simple eyes, known as ocelli, which aid in attitude control, horizon detection, and in detecting changes in the polarized skylight pattern.^20^^,^^21^ Across all ants, nocturnal species typically have larger lenses and wider photoreceptors to capture more light.^18^^,^^22^^,^^23^^,^^24^ Caste- and sex-specific differences were also found in external antennal morphology, critical for chemical communication.^25^ In *Camponotus japonicus*, alate females had the most sensilla and males had the least.^26^ While certain types of sensilla were common across all castes, their distribution on the antennae differed significantly, with one type of sensilla (basiconica sensillum) noticeably absent in male antennae.^26^

**Figure 1:**
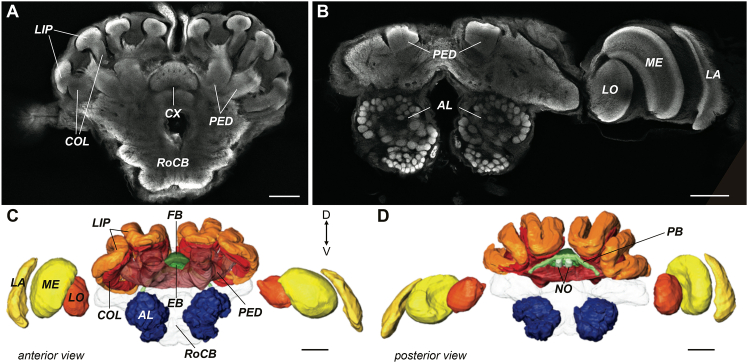
General layout of the ant brain (A and B) Frontal sections of a *M. brevinoda* alate female brain labeled with anti-synapsin highlighting the major neuropils included in this study: the optic lobes (OL: lamina [LA], medulla [ME], lobula [LO]), antennal lobes (AL), mushroom bodies (MB: lip [LIP], collar [COL], peduncle [PED]), central complex (CX: fan-shaped body [FB], ellipsoid body [EB], protocerebral bridge [PB], noduli [NO]), and the rest of the central brain (RoCB) (C) Anterior and (D) posterior views of surface reconstruction of neuropils from the same individual in (A and B). Scale bars, 200 μm. See Figure S1 for example brains confocal scans and reconstruction of the brain of alate female of *Rhytidoponera metallica*.

Information captured from the external sensory structures is first processed by primary sensory neuropils, the optic lobes and antennal lobes (abbreviations in Table 1). Visual input is processed by the subregions within the optic lobe: the lamina, medulla, and lobula, while olfactory information is transmitted to the antennal lobe. Information from these sensory neuropils is received by higher-order, information-integrating regions, including the mushroom body, which receives inputs from optic lobe and antennal lobe in the collar and lip regions, respectively, and relays it to the surrounding protocerebrum via the peduncle and lobes. The central complex is the main navigation center, with four subregions (the fan-shaped body [FB], the ellipsoid body [EB], the protocerebral bridge, and the noduli) that encode internal representations of body orientation from sensory input (Figure 1; Figure S1). The volume of the neuropils provides a glimpse into the relative investment an animal has made into processing a specific type of sensory information, supporting associative memory, or mediating sensorimotor transformations. Nocturnal hawkmoths and paper wasps, for example, invested more in their antennal lobes compared to optic lobes, suggesting less reliance on visual information.^27^^,^^28^ Cave beetles that spend their entire lives in complete darkness have evolved to lose their visual processing centers altogether.^29^ Among worker ants of *Myrmecia* active at discrete times of the day, nocturnal ants invested less in primary visual processing neuropils and more in primary olfactory processing regions and downstream neuropils that integrate visual and olfactory information.^30^

**Table 1:** List of abbreviations of all neuropils mapped in this study

Abbreviation	Neuropil name
LA	lamina
ME	medulla
LO	lobula
OL	optic lobe
AL	antennal lobe
LIP	calyx lip
COL	calyx collar
PED	peduncle and lobes
MB	mushroom body
FB	fan-shaped body
EB	ellipsoid body
PB	protocerebral bridge
NO	noduli
CX	central complex
RoCB	rest of central brain

In this study, we aimed to identify the differential investment in brain regions between distinct castes and sexes in two solitary foraging Australian ants, *Myrmecia brevinoda* and *Rhytidoponera metallica*. We chose to address this question in two visually oriented ants of the genus *Myrmecia* (subfamily Myrmeciinae) and *Rhytidoponera* (subfamily Ectatomminae), since these two represent early diverging formicoid ant lineages, making them key taxa for examining the evolution of morphological and behavioral traits. Workers, alate females and alate males exhibit clear differences in locomotory strategies and lifestyle. Workers of both species are exclusively pedestrian, forage individually using visual information, and engage in nest maintenance activities. The behavior of the alates between the two species varied. *Rhytidoponera metallica* produces gamergates, where workers exhibit a calling behavior during which pheromones are released from pygidial glands.^31^ In contrast, in *Myrmecia brevinoda,* alate males and females leave the nest independently, climb trees and fly toward the nearby hilltops, like the related *Myrmecia pyriformis* (pers. obs. A.N.). At these hilltops, they form a large ball where females are thought to produce odor cues to attract males. Following mating, a mated female establishes a new colony and forages until the first batch of workers start foraging. In addition, both species exhibited dramatic variation in body size and in their activity times. Workers of *M. brevinoda* are strictly nocturnal, but the winged males and females fly out during the day, which leads to distinct activity times of castes within a single species (pers. obs. A.N.; see also^18^). In contrast, in *R. metallica*, all three castes are strictly day-active. Workers of *M. brevinoda* are more than twice the size of *R. metallica*. Workers of both species use visual cues for navigation and obtain compass information from celestial cues.^32^^,^^33^ Young queens of both species (*R. metallica*^34^; *M. brevinoda*,^16^ pers. obs. A.N.) also spend a brief period in their life foraging while establishing new nests. We analyzed the brains of workers, alate females and males in both ant species and carried out a scaling analysis of distinct neuropils. We predict that in both species, the males, which are exclusive fliers and non-foragers, will have the largest investment into optic lobes and the least in mushroom bodies. Between species we expect that in each caste *Myrmecia* ants will have the largest investment in all brain regions that receive visual input.

## Results

### Variation in total neuropil volumes

**Figure 2:**
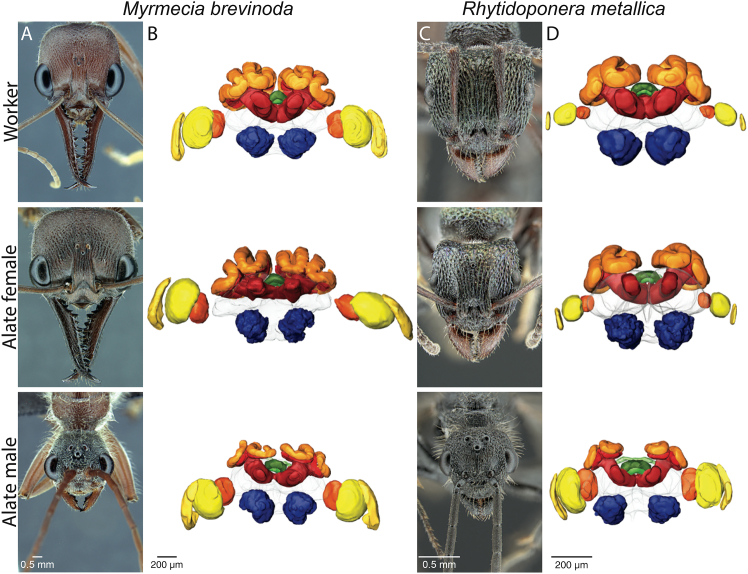
Comparative head and brain morphology of three castes in *Myrmecia brevinoda* and *Rhytidoponerametallica* (A and C) Example images of the head of a worker (top), an alate female (middle) and a male (bottom) ant to show variation in size and head morphology between castes in *M. brevinoda* and *R. metallica*, respectively. (B and D) Representative surface renderings of a worker (top), an alate female (middle) and a male (bottom) brain in *M. brevinoda* and *R. metallica*, respectively.

Both the head width and total neuropil volume varied between all three castes of *M. brevinoda* and *R. metallica* (Figure 2; Figure S2). Across all three castes, *M. brevinoda* had a greater head width compared to *R. metallica*. Within each species, males consistently had the smallest head size, and alate females were the largest or comparable to workers (Table 2; Table S1). We observed a similar trend in the total absolute neuropil volume. Across all three castes, individuals of *M. brevinoda* had larger total neuropil volume compared to *R. metallica* (by an order of magnitude), which correlated with their head widths (Figure S2). In both species, males had the smallest total neuropil volume while alate females and workers were similar (Table 3; Table S1).

**Table 2:** Comparison of head width between workers, alate females and alate males in *Myrmecia brevinoda* and *Rhytidoponera metallica*

		Head width (mm, mean ± s.d)	Kruskal-Wallis statistics	Dunn’s post-hoc test
*M. brevinoda*	workers (*n* = 28)	3.45 ± 0.5	χ^2^ = 16.192 *p* = 0.0003	alate males vs. alate females *p* = 0.0001
	alate females (*n* = 3)	4.60 ± 0.07		alate females vs. workers *p* = 0.0205
	alate males (*n* = 5)	2.61 ± 0.09		workers vs. alate males *p* = 0.0064
*R. metallica*	workers (*n* = 6)	1.53 ± 0.12	χ^2^ = 11.3072 *p* = 0.0035	alate males vs. alate females *p* = 0.0013
	alate females (*n* = 6)	1.63 ± 0.07		alate females vs. workers *p* = 0.3794
	alate males (*n* = 5)	1.24 ± 0.06		workers vs. alate males *p* = 0.0382

**Table 3:** Comparison of total neuropil volume between workers, alate females and alate males in *Myrmecia brevinoda* and *Rhytidoponera metallica*

		Total neuropil volume (μm^3^, mean ± s.d)	Kruskal-Wallis statistics	Dunn’s post-hoc test
*M. brevinoda*	workers (*n* = 28)	3.41 × 10^8^ ± 5.07 × 10^7^	χ^2^ = 8.2875 *p* = 0.0159	alate males vs. alate females *p* = 0.0394
	alate females (*n* = 6)	3.67 × 10^8^ ± 1.53 × 10^8^		alate females vs. workers *p* = 1.0
	alate males (*n* = 6)	2.49 × 10^8^ ± 2.60 × 10^7^		workers vs. alate males *p* = 0.007
*R. metallica*	workers (*n* = 7)	3.97 × 10^7^ ± 1.09 × 10^7^	χ^2^ = 8.9163 *p* = 0.0116	alate males vs. alate females *p* = 0.0088
	alate females (*n*-7)	4.25 × 10^7^ ± 3.88 × 10^6^		alate females vs. workers *p* = 1.0
	alate males (*n* = 6)	2.37 × 10^7^ ± 1.16 × 10^7^		workers vs. alate males *p* = 0.0213

### Optic lobes

The optic lobes, comprising the lamina, medulla, and lobula, are the primary visual processing centers in the insect brain (Figure 1). We first compared the scaling pattern of optic lobe of each caste between species.

#### Comparison between species

**Figure 3:**
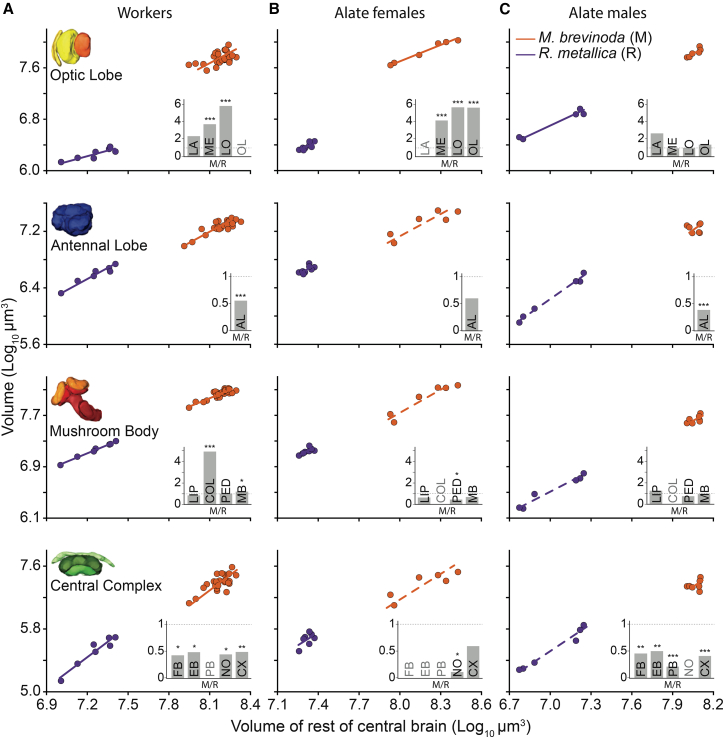
Scaling relationship of the volume of the major neuropils and the sub-neuropils between the two species, *Myrmecia brevinoda* and *Rhytidoponera metallica* (A–C) Comparison of scaling relations of the main neuropils to the reference structure, RoCB, between the two study species, *M. brevinoda* (orange) and *R. metallica* (purple), across workers, alate females and alate males are shown. Each representation shows comparisons between species for the same neuropil. Regression lines are derived from SMA analysis and fitted to the data. Dashed lines represent non-significant linear regression models. The inset shows gsi for the region of interest when comparing both species. The gsi estimates the difference in neuropil size of the same caste between two species, calculated using the elevation of the regression line. From left to right, bars represent gsi for each sub-neuropil and the whole neuropil when comparing *M. brevinoda* (M) to *R. metallica* (R). A gsi of 1 indicates that both species have a similarly sized neuropil, shown by the horizontal dotted line. When comparing both species, a gsi <1 indicates that the neuropil is larger in *R. metallica* than in *M. brevinoda* in the comparison and vice versa. In cases where it was theoretically invalid to test for volumetric differences, no gsi was calculated (indicated by gray text). Stars indicate the significance level of difference in elevation between two regression lines, such that ∗*p* < 0.05, ∗∗*p* < 0.01 and ∗∗∗*p* < 0.001.

In worker ants that are strictly pedestrian, the optic lobe volume scaled differently in both *M. brevinoda* and *R. metallica*, making it theoretically invalid to test for differences in a grade shift (Figure 3A, top panel). However, each of the three sub-neuropils of the optic lobe scaled similarly, i.e., their slopes were not significantly different and thus could be compared for differences in volume. The lamina did not differ significantly between workers of the two species. However, relative to the reference structure, the RoCB, both the medulla and lobula, were significantly larger in the workers of *M. brevinoda* compared to *R. metallica* (Figure 3A, top panel inset).

In the alate females that lead a walking and flying lifestyle, relative to the RoCB, the optic lobes were larger in *M. brevinoda* compared to *R. metallica* (Figure 3B, top panel). A volumetric difference was also found in the medulla and lobula, while the lamina exhibited distinct scaling patterns in both species (Figure 3B, top panel inset).

Males that lead a life exclusively on the wing exhibited no significant differences in the volume of the optic lobes (Figure 3C, top panel) and all sub-neuropils relative to the RoCB between the two species (Figure 3C, top panel inset; Table 4).

**Table 4:** Outputs of standardized major axis regression on log transformed data (log y = α + βlog x) comparing the scaling relationships of each neuropil with the volume of the reference structure, the RoCB, between the two species, *Myrmecia brevinoda* and *Rhytidoponera metallica*

Caste	y	Do groups have a common slope? H0 = slopes are equal	Is the common slope different from 1 H0 = common slope not different from one	Is the linear model (lm) for *M. brevinoda* significant?	Is the linear model (lm) for *R. metallica* significant?	Are there differences in elevation? H0: no difference in elevation	α_*M.brevinoda*_	α_*R. metallica*_	gsi (*M. brevinoda* “M”/*R.metallica* “R”)
Workers	LA	yes *p* = 0.73	no *p* = 0.08	no *p* = 0.21	no *p* = 0.53	no *p* = 0.21	−5.17	−5.52	2.21
	ME	yes *p* = 0.15	no *p* = 0.37	yes *p* = 0.01	yes *p* = 0.02	yes *p* = 4.42 × 10^−6^	−0.24	−0.8	3.62
	LO	no *p* = 0.05	no *p* = 0.15	yes *p* = 0.01	yes *p* = 0.01	yes *p* = 6.78 × 10^−12^	−0.2	−0.96	5.76
	OL	no *p* = 0.03	–	yes *p* = 0.02	yes *p* = 0.01	–	–	–	–
	AL	yes *p* = 0.26	no *p* = 0.41	yes *p* < 0.01	yes *p* < 0.01	yes *p* = 0.41 × 10^−3^	−1.3	−1.03	0.53
	LIP	yes *p* = 0.74	no *p* = 0.79	yes *p* < 0.01	yes *p* < 0.01	no *p* = 0.33	0.009	0.09	0.84
	COL	yes *p* = 0.45	no *p* = 0.26	yes *p* < 0.01	no *p* = 0.30	yes *p* = 7.50 × 10^−12^	0.48	−0.22	4.93
	PED	yes *p* = 0.85	no *p* = 0.61	yes *p* < 0.01	yes *p* < 0.01	no *p* = 0.97	0.002	-4E−04	1.01
	MB	yes *p* = 0.94	yes *p* = 0.03	yes *p* < 0.01	yes *p* < 0.01	yes *p* = 0.02	0.92	0.85	1.19
	FB	no *p* = 0.05	yes *p* = 0.02	yes *p* < 0.01	yes *p* = 0.01	yes *p* = 0.01	−5.4	−5.02	0.41
	EB	yes *p* = 0.39	no *p* = 0.05	yes *p* < 0.01	yes *p* = 0.02	yes *p* = 0.04	−5.71	−5.38	0.47
	PB	no *p* = 0.001	–	yes *p* < 0.01	yes *p* = 0.01	–	–	–	–
	NO	yes *p* = 0.14	no *p* = 0.08	no *p* = 0.07	yes *p* = 0.01	yes *p* = 0.02	−4.99	−4.62	0.43
	CX	yes *p* = 0.43	no *p* = 0.06	yes *p* < 0.01	yes *p* < 0.01	yes *p* = 0.005	−4.27	−3.95	0.48
Alate females	LA	no *p* = 0.02	–	yes *p* = 0.01	no *p* = 0.34	–	–	–	–
	ME	yes *p* = 0.05	no *p* = 0.13	yes *p* < 0.01	yes *p* = 0.01	yes *p* = 4.5 × 10^−14^	0.1	−0.5	4.05
	LO	yes *p* = 0.36	no *p* = 0.22	yes *p* < 0.01	no *p* = 0.06	yes p = < 2.22 × 10^−16^	0.28	−0.46	5.57
	OL	yes *p* = 0.07	no *p* = 0.09	yes *p* < 0.01	yes *p* = 0.02	yes p = < 2.22 × 10^−16^	0.9	0.16	5.53
	AL	yes *p* = 0.31	no *p* = 0.57	yes *p* = 0.04	no *p* = 0.07	no *p* = 0.18	−1.25	−1	0.57
	LIP	yes *p* = 0.43	no *p* = 0.49	yes *p* = 0.01	no *p* = 0.55	no *p* = 0.15	−1.94	−1.68	0.55
	COL	no *p* = 0.03	–	yes *p* < 0.01	no *p* = 0.12	–	–	–	–
	PED	yes *p* = 0.22	no *p* = 0.05	yes *p* = 0.01	yes *p* = 0.01	yes *p* = 0.01	−4	−3.55	0.36
	MB	yes *p* = 0.91	no *p* = 0.48	yes *p* < 0.01	no *p* = 0.06	no *p* = 0.13	−1.86	−1.64	0.6
	FB	no *p* = 0.02	–	yes *p* = 0.02	no *p* = 0.22	–	–	–	–
	EB	no *p* = 0.04	–	yes *p* = 0.01	no *p* = 0.11	–	–	–	–
	PB	no *p* = 0.01	–	no *p* = 0.27	yes *p* = 0.03	–	–	–	–
	NO	yes *p* = 0.05	yes *p* = 0.04	no *p* = 0.26	no *p* = 0.36	yes *p* = 0.04	−10.18	−9.17	0.1
	CX	yes *p* = 0.05	no *p* = 0.13	yes *p* = 0.03	no *p* = 0.17	no *p* = 0.29	−2.55	−2.31	0.58
Alate males	LA	yes *p* = 0.36	no *p* = 0.65	no *p* = 0.13	no *p* = 0.16	no *p* = 0.13	−1.02	−1.43	2.54
	ME	yes *p* = 0.1	no *p* = 0.22	yes *p* = 0.02	yes *p* < 0.01	no *p* = 0.81	−0.93	−0.9	0.95
	LO	yes *p* = 0.13	no *p* = 0.30	no *p* = 0.17	yes *p* < 0.01	no *p* = 0.87	−0.49	−0.51	1.05
	OL	yes *p* = 0.12	no *p* = 0.29	yes *p* = 0.03	yes *p* = 0.01	no *p* = 0.44	−0.12	−0.2	1.22
	AL	yes *p* = 0.89	yes *p* = 0.02	no *p* = 0.95	yes *p* < 0.01	yes *p* = 2.26 × 10^−7^	−3.65	−3.21	0.36
	LIP	yes *p* = 0.19	no *p* = 0.43	no *p* = 0.89	yes *p* < 0.01	no *p* = 0.59	−0.84	−0.92	1.19
	COL	no *p* = 0.03	–	yes *p* = 0.03	yes *p* < 0.01	–	–	–	–
	PED	yes *p* = 0.42	no *p* = 0.13	no *p* = 0.05	yes *p* < 0.01	no *p* = 0.11	−2.43	−2.26	0.68
	MB	yes *p* = 0.56	no *p* = 0.6	no *p* = 0.17	yes *p* < 0.01	no *p* = 0.72	−1.17	−1.13	0.91
	FB	yes *p* = 0.34	no *p* = 0.27	no *p* = 0.45	yes *p* < 0.01	yes *p* = 0.001	−2.99	−2.65	0.45
	EB	yes *p* = 0.11	no *p* = 0.15	no *p* = 0.23	yes *p* < 0.01	yes *p* = 0.006	−3.37	−3.07	0.5
	PB	yes *p* = 0.07	yes *p* = 0.02	no *p* = 0.22	yes *p* = 0.01	yes *p* = 1.68 × 10^−4^	−5.22	−4.54	0.21
	NO	no *p* = 0.01	–	no *p* = 0.22	yes *p* = 0.01	–	–	–	–
	CX	yes *p* = 0.63	no *p* = 0.33	no *p* = 0.44	yes *p* < 0.01	yes *p* = 4.68 × 10^−4^	−2.96	−2.57	0.41

Abbreviations and gsi conventions are described in ‘STAR Methods’.

#### Comparison within species

**Figure 4:**
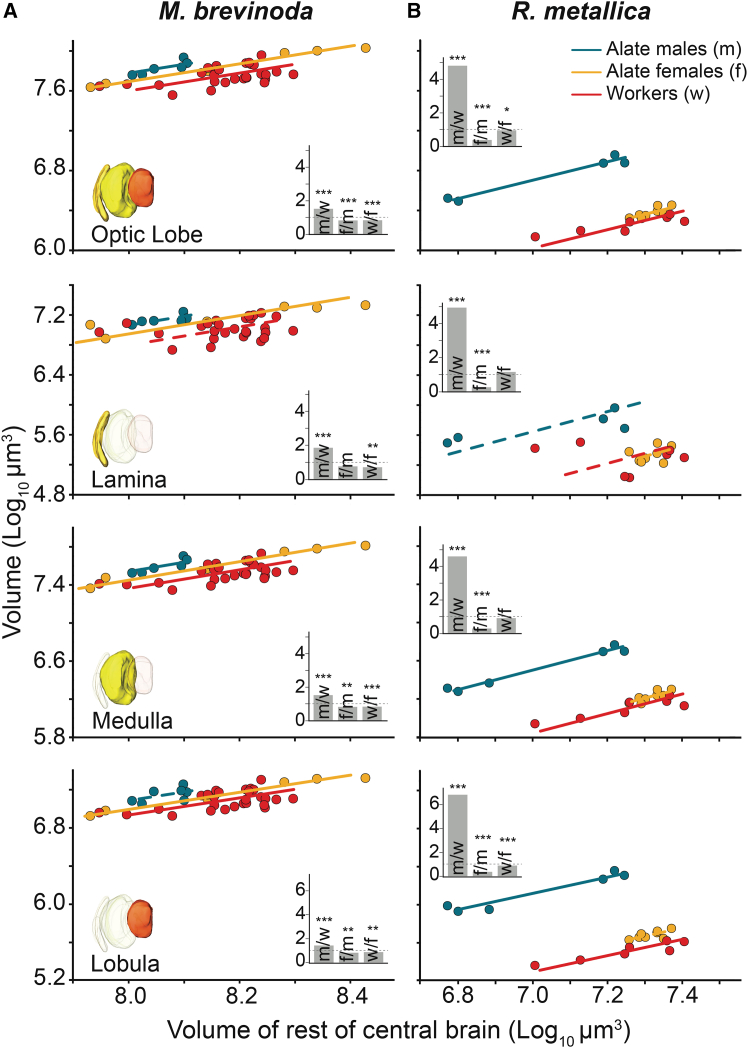
Scaling relationship of the volume of the optic lobes and the sub neuropils between the castes of *Myrmecia brevinoda* and *Rhytidoponera metallica* Scaling relations of the whole optic lobe, lamina, medulla, and lobula to the reference structure, the RoCB, for alate males (blue), alate females (yellow), and workers (red) of *M. brevinoda* (A) and *R. metallica* (B). Each representation shows comparisons between castes of the same species. Regression lines are derived from SMA analysis and fitted to the data. Dashed lines represent non-significant linear regression models. The inset for each graph shows gsi for the region of interest. The gsi estimates the difference in the neuropil size between two castes of the same species and is calculated using the elevation of the regression line. From left to right, bars represent gsi calculated for alate males and workers (m/w), alate females and alate males (f/m) and workers and alate females (w/f). A gsi of 1 indicates that both castes have a similarly sized neuropils, shown by the horizontal dotted line within the bar graph. A gsi <1 indicates that the neuropil is larger in the second caste in the comparison and vice versa. If scaling slopes significantly differed between the three groups being compared, rendering it theoretically invalid to test for a true difference in volume/grade shift, no gsi was calculated (indicated by a missing inset). Stars indicate the significance level of difference in elevation between two regression lines, such that ∗*p* < 0.05, ∗∗*p* < 0.01 and ∗∗∗*p* < 0.001.

We compared the scaling relationships of neuropil volumes of the three castes within each species. In both *M. brevinoda* and *R. metallica*, males exhibited significantly larger optic lobe volumes relative to the RoCB than either of the female castes. In both species, between the two female castes, the alates possessed relatively larger optic lobes compared to the workers (Figures 4A and 4B, top panel). The magnitude of this difference between males and either of the female castes was more pronounced in *R. metallica* than in *M. brevinoda* (Figure 4B, top panel inset). Male ants, thus, have the largest optic lobe neuropil volume, while workers have the smallest, relative to RoCB. This pattern was consistent across all sub-neuropils of the optic lobe (Figure 4), with few variations. For instance, in *M. brevinoda*, males and alate females had comparable lamina volumes (Figure 4A, second panel inset), while in *R. metallica*, alate females and workers shared similar lamina and medulla volumes (Figure 4B, second and third panel insets; Table 5).

**Table 5:** Outputs of standardized major axis regression on log transformed data (log y = α + βlog x) comparing the scaling relationships of each neuropil with the volume of the reference structure, the RoCB, between the workers, alate males (males), and alate females (females) of *Myrmecia brevinoda* and *Rhytidoponera metallica*

Species	Region of interest	Do groups have a common slope? H0 = slopes are equal	Pairwise difference in slope Does slope of caste 1 differ from slope of caste 2? H0 = no difference in elevation			Is the common slope different from 1? H0 = common slope not different from 1	Is the linear model significant? (*p*-values) Yes, *p* < 0.05 No, *p* > 0.05	Are there differences in elevation? H0: no difference in elevation	Pairwise difference in elevation Does elevation of caste 1 differ from elevation of caste 2? H0 = no difference in elevation			α_males_	α_Workers_	α_Females_	gsi (m/w)	gsi (f/m)	gsi (w/f)
			Caste 1	Caste 2	*p value*				Caste 1	Caste 2	*p value*						
*M. brevinoda*	LA	yes *p* = 0.16				no *p* = 0.11	males = 0.13 workers = 0.21 females = 0.01	yes *p* = 2.90 × 10^−11^	males	workers	8.71 × 10^−11^	−2.72	−2.99	−2.84	1.84	0.77	0.71
									females	males	0.07						
									workers	females	0.009						
	ME	yes *p* = 0.08				no *p* = 0.18	males = 0.02 workers = 0.01 females <0.01	yes *p* = 8.37 × 10^−12^	males	workers	2.03 × 10^−12^	−0.24	−0.42	−0.33	1.5	0.81	0.82
									females	males	0.002						
									workers	females	1.87 × 10^−4^						
	LO	yes *p* = 0.08				no *p* = 0.16	males = 0.17 workers = 0.01 females <0.01	yes *p* = 9.68 × 10^−7^	males	workers	1.53 × 10^−7^	−0.09	−0.24	−0.18	1.42	0.81	0.87
									females	males	0.002						
									workers	females	0.005						
	OL	yes *p* = 0.06				no *p* = 0.12	males = 0.03 workers = 0.02 females <0.01	yes *p* = 1.01 × 10^−12^	males	workers	8.68 × 10^−13^	0.66	0.49	0.58	1.51	0.82	0.81
									females	males	3.48 × 10^−4^						
									workers	females	3.56 × 10^−6^						
*R. metallica*	LA	yes *p* = 0.12				no *p* = 0.13	males = 0.16 workers = 0.53 females = 0.34	yes *p* = 3.55 × 10^−7^	males	workers	7.12 × 10^−6^	−3.73	−4.42	−4.46	4.91	0.19	1.09
									females	males	7.89 × 10^−7^						
									workers	females	0.64						
	ME	yes *p* = 0.13				no *p* = 0.25	males <0.01 workers = 0.02 females = 0.01	yes *p* < 2.22 × 10^−16^	males	workers	<2.22 × 10^−16^	−0.65	−1.31	−1.25	4.57	0.25	0.87
									females	males	<2.22 × 10^−16^						
									workers	females	0.09						
	LO	yes *p* = 0.33				no *p* = 0.26	males <0.01 workers = 0.01 females = 0.06	yes *p* < 2.22 × 10^−16^	males	workers	<2.22 × 10^−16^	0.17	−0.66	−0.54	6.66	0.2	0.76
									females	males	<2.22 × 10^−16^						
									workers	females	2.26 × 10^−8^						
	OL	yes *p* = 0.06				no *p* = 0.11	males = 0.01 workers = 0.01 females = 0.02	yes *p* < 2.22 × 10^−16^	males	workers	<2.22 × 10^−16^	0.24	−0.44	−0.38	4.8	0.24	0.87
									females	males	<2.22 × 10^−16^						
									workers	females	0.02						

Species	ROI	Common slope?	Pairwise difference in slope			Common slope different from 1?	Is the linear model significant?	Are there differences in elevation?	Pairwise difference in elevation			α_males_	α_Workers_	α_Females_	gsi (m/w)	gsi (f/m)	gsi (w/f)
*M. brevinoda*	AL	yes *p* = 0.6				no *p* = 0.49	males = 0.95 workers <0.01 females = 0.04	yes *p* = 0.04	males	workers	0.01	−1.71	−1.79	−1.75	1.22	0.9	0.91
									females	males	0.56						
									workers	females	0.43						
*R.metallica*	AL	yes *p* = 0.11				yes *p* = 0.03	males <0.01 workers <0.01 females = 0.07	no *p* = 0.97				−2.94	−2.94	−2.94	0.98	1	1.02
*M. brevinoda*	LIP	yes *p* = 0.4				no *p* = 0.59	males = 0.89 workers <0.01 females = 0.01	yes *p* = 9.99 × 10^−16^	males	workers	<2.22 × 10^−16^	−1.25	−0.92	−0.97	0.47	1.92	1.11
									females	males	1.13 × 10^−5^						
									workers	females	0.27						
	COL	no *p* = 0.002	males	workers	0.005		males = 0.03 workers <0.01 females <0.01										
			females	males	0.08												
			workers	females	0.02												
	PED	yes *p* = 0.13				no *p* = 0.26	males = 0.05 workers <0.01 females = 0.01	yes *p* < 2.22 × 10^−16^	males	workers	<2.22 × 10^−16^	−0.98	−0.74	−0.88	0.58	1.27	1.37
									females	males	0.13						
									workers	females	0.002						
	MB	yes *p* = 0.21				no *p* = 0.33	males = 0.17 workers <0.01 females <0.01	yes *p* < 2.22 × 10^−16^	males	workers	<2.22 × 10^−16^	−0.22	0.07	−0.01	0.51	1.63	1.21
									females	males	5.07 × 10^−5^						
									workers	females	0.04						
*R. metallica*	LIP	yes *p* = 0.46				no *p* = 0.64	males <0.01 workers <0.01 females = 0.55	yes *p* < 2.22 × 10^−16^	males	workers	<2 × 10^−16^	−0.61	−0.06	−0.12	0.28	3.1	1.15
									females	males	<2 × 10^−16^						
									workers	females	0.03						
	COL	no *p* = 0.01	males	workers	0.2		males <0.01 workers = 0.03 females = 0.12										
			females	males	0.009												
			workers	females	0.004												
	PED	yes *p* = 0.11				no *p* = 0.07	males <0.01 workers <0.01 females = 0.01	yes *p* = 2.22 × 10^−15^	males	workers	<2.22 × 10^−16^	−2.04	−1.73	−1.84	0.49	1.59	1.28
									females	males	2.81 × 10^−6^						
									workers	females	3.56 × 10^−4^						
	MB	yes *p* = 0.16				no *p* = 0.09	males <0.01 workers <0.01 females = 0.06	yes *p* < 2.22 × 10^−16^	males	workers	<2.22 × 10^−16^	0.28	0.7	0.62	0.38	2.21	1.18
									females	males	2.35 × 10^−12^						
									workers	females	6.48 × 10^−8^						

Species	ROI	Common slope?	Pairwise difference in slope			Common slope different from 1?		Are there differences in elevation?	Pairwise difference in elevation			α_males_	α_Workers_	α_Females_	gsi (m/w)	gsi (f/m)	gsi (w/f)
*M. brevinoda*	FB	yes *p* = 0.19				no *p* = 0.24	males = 0.45 workers <0.01 females = 0.02	yes *p* = 0.01	males	workers	0.002	−2.8	−2.9	−2.92	1.27	0.75	1.05
									females	males	0.05						
									workers	females	0.67						
	EB	yes *p* = 0.3				no *p* = 0.1	males = 0.24 workers <0.01 females = 0.01	yes *p* = 0.01	males	workers	0.03	−4.31	−4.39	−4.46	1.18	0.71	1.2
									females	males	0.004						
									workers	females	0.03						
	PB	yes *p* = 0.14				yes *p* = 0.001	males = 0.22 workers <0.01 females = 0.27	no *p* = 0.64	males	workers	0.74	−8.07	−8.12	−8.12	1.12	0.88	1.01
									females	males	0.22						
									workers	females	0.97						
	NO	yes *p* = 0.05				yes *p* = 0.003	males = 0.22 workers = 0.07 females = 0.26	yes *p* = 0.02	males	workers	0.003	−7.93	−8.22	−8.21	1.96	0.52	0.98
									females	males	0.09						
									workers	females	0.93						
	CX	yes *p* = 0.4				no *p* = 0.4	males = 0.44 workers <0.01 females = 0.03	yes *p* = 0.02	males	workers	0.004	−2.68	−2.77	−2.8	1.23	0.77	1.06
									females	males	0.09						
									workers	females	0.65						
*R. metallica*	FB	no *p* = 0.03	males	workers	0.02		males <0.01 workers = 0.01 females = 0.22										
			females	males	0.09												
			workers	females	0.69												
	EB	no *p* = 0.04	males	workers	0.13		males <0.01 workers = 0.02 females = 0.11										
			females	males	0.03												
			workers	females	0.26												
	PB	no *p* = 0.003	males	workers	0.08		males <0.01 workers = 0.01 females = 0.04										
			females	males	0.007												
			workers	females	0.0006												
	NO	yes *p* = 0.06				yes *p* = 0.04	males = 0.01 workers = 0.01 females = 0.36	yes *p* = 7.76 × 10^−10^	males	workers	2.93 × 10^−10^	−4.39	−4.81	−4.71	2.67	0.48	0.79
									females	males	1.42 × 10^−4^						
									workers	females	0.06						
	CX	yes *p* = 0.2				yes *p* = 0.03	males <0.01 workers <0.01 females = 0.12	yes *p* = 6.01 × 10^−14^	males	workers	6.77 × 10^−15^	−3.23	−3.55	−3.47	2.1	0.57	0.84
									females	males	1.22 × 10^−6^						
									workers	females	0.11						

Abbreviations and gsi conventions are described in ‘STAR Methods’.

### Antennal lobe

The antennal lobes (ALs) are the primary olfactory processing regions (Figure 1).

#### Comparison between species

Workers and males of *R. metallica* had larger antennal lobes relative to the RoCB compared to their counterparts in *M. brevinoda* (Figures 3A and 3C, second row panels). In alate females, antennal lobe volumes were relatively larger in *R. metallica*; however, they were not significantly bigger than the alate females of *M. brevinoda* (Figure 3B, second row panel; Table 4).

#### Comparison within species

**Figure 5:**
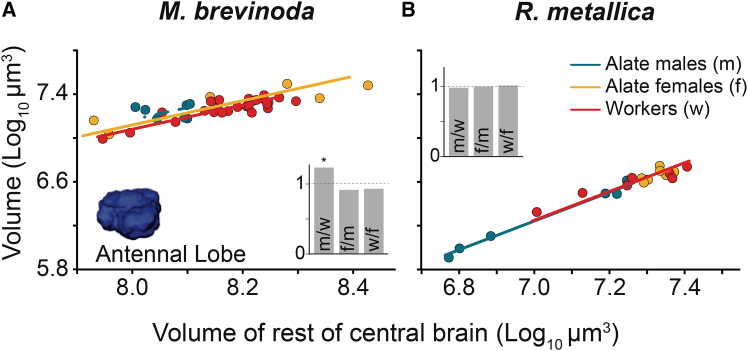
Scaling relationship of the volume of the antennal lobe between the castes of *Myrmecia brevinoda* and *Rhytidoponera metallica* Scaling relations of the AL to the reference structure, the RoCB, for alate males (blue), alate females (yellow), and workers (red) of *M. brevinoda* (A) and *R. metallica* (B). Figure conventions are as described in Figure 4.

The antennal lobes showed caste-specific differences in volume only in *M. brevinoda*. With respect to the RoCB volume, males had relatively larger antennal lobes than workers. However, alate females did not differ significantly from males or workers (Figure 5A and inset within). In *R. metallica*, however, antennal lobe volumes did not vary significantly across castes (Figure 5B; Table 5).

### Mushroom bodies

The mushroom bodies are the site of learning and memory in the insect brain. We divided the mushroom body into three sub-neuropils: the lip, the collar, and the peduncle (which also includes the output regions of the mushroom body; Figure 1).

#### Comparison between species

Within workers, relative to the RoCB, mushroom bodies were significantly larger in *M. brevinoda* than *R. metallica* (Figure 3A, third row panel). This difference was due to the significantly larger collar region (almost four times larger) in *M. brevinoda*, while the lip and peduncle were similar in size between the workers of the two species (Figure 3A, third row panel inset). Among alate females and males, the overall mushroom body volume was comparable between species. In both castes, the lip showed no notable differences and due to distinct scaling patterns, the collar could not be compared for absolute volume differences. Only in alate females, the peduncle was larger in *R. metallica* compared to *M. brevinoda* (Figure 3B, third row panel inset; Table 4).

Lastly, relative to the optic lobe, males and females of *R. metallica* showed significantly larger collar region compared to *M. brevinoda*. Workers, on the other hand, were comparable between the two species. However, relative to the antennal lobe, in both males and workers, the lip was larger in *M. brevinoda* compared to *R. metallica*. When comparing the collar between both species, with the lip as the reference structure, it was seen that females and workers of *M. brevinoda* showed significantly larger collar regions when compared to *R. metallica*, while *R. metallica* males had larger collar regions than *M. brevinoda* males (Table S2).

#### Comparison within species

**Figure 6:**
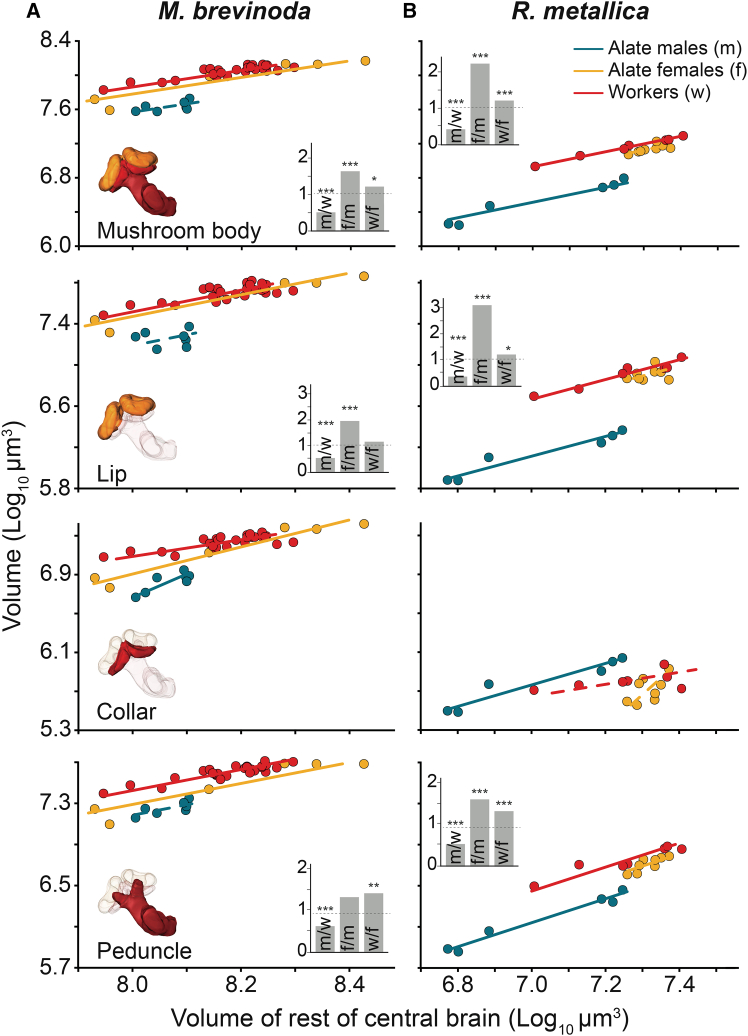
Scaling relationship of the volume of the mushroom body and the constituent neuropils between the castes of *Myrmecia brevinoda* and *Rhytidoponera metallica* Scaling relations of the MB and its constituent neuropils to the reference structure, the RoCB, for alate males (blue), alate females (yellow), and workers (red) of *M. brevinoda* (A) and *R. metallica* (B). Figure conventions are as described in Figure 4.

In both *M. brevinoda* and *R. metallica*, relative to the RoCB, males consistently had the smallest mushroom bodies, alate females exhibited intermediate mushroom body sizes, and workers possessed the largest. The magnitude of the volumetric difference was larger between alate female and males than between the two female castes (Figures 6A and 6B top panel and inset). The general trend of mushroom body size—with workers having the largest and males the smallest—was consistent across both the lip and peduncle (Figures 6A and 6B, second and fourth row panels), with two exceptions: alate females and workers had similarly sized lip regions and alate females and males had similarly sized peduncle in *M. brevinoda* (Figure 6A, second and fourth row panel insets). Scaling slopes of collar volumes among the three castes differed significantly in both species, rendering it theoretically invalid to test for a difference in volume or grade shift (Figures 6A and 6B third row panels; Table 5).

We compared the collar and lip with their functionally connected primary sensory regions, optic lobe and antennal lobe, respectively (Table S3). Relative to the optic lobe, the collar scaled differently between the three castes of *M. brevinoda* and thus could not be tested for true differences in volume. On the other hand, relative to antennal lobe, workers had the largest and males the smallest lip regions. Lastly, when comparing the scaling of the collar to the lip (reference structure), there were no significant differences between all three castes of *M. brevinoda*.

Within the castes of *R. metallica*, workers consistently had the largest COL and LIP regions, followed by females, while males had the smallest, when comparing the COL with OL as the reference structure and the LIP with the AL as the reference structure. Lastly, males of *R. metallica* possessed significantly larger COL regions when compared to either of the female castes, in reference to the LIP region (Table S3).

### Central complex

The central complex (CX) is a group of four neuropils: the FB, EB, protocerebral bridge, and the paired noduli (Figure 1; Figure S1).

#### Comparison between species

Workers of *R. metallica* showed significantly larger central complexes relative to the RoCB, when compared to their *M. brevinoda* counterparts (Figure 3A, bottom panel). This trend was seen in all sub-neuropils of the central complex in the workers, except the protocerebral bridge, where scaling differed between the two species and thus could not be compared for grade shifts (Figure 3A, bottom panel inset). In alate females, the overall volumes of the central complex, with the exception of the noduli, were not significantly different (Figure 3B, bottom panel). The FB, EB, protocerebral bridge showed varying scaling patterns in alate females (Figure 3B, bottom panel inset). Lastly, in males, the central complex of *R. metallica* was significantly larger relative to the RoCB compared to *M. brevinoda* (Figure 3C, bottom panel). This pattern was seen in all neuropils of the central complex, except the noduli, which scaled differently in both species (Figure 3C; Table 4).

#### Comparison within species

**Figure 7:**
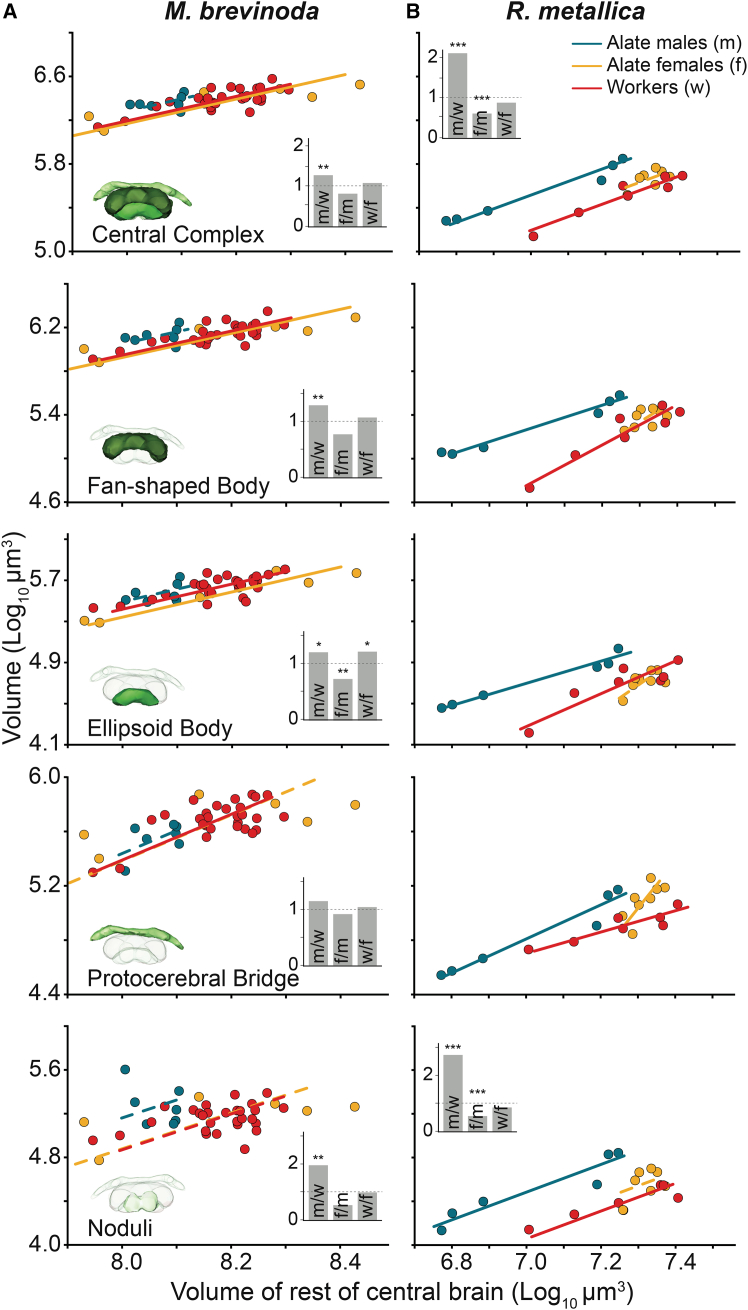
Scaling relationship of the volume of the central complex and the constituent neuropils between the castes of *Myrmecia brevinoda* and *Rhytidoponera metallica* Scaling relations of the CX and its constituent neuropils to the reference structure, the RoCB, for alate males (blue), alate females (yellow) and workers (red) of *M. brevinoda* (A) and *R. metallica* (B). Figure conventions are as described in Figure 4.

In both species, relative to the RoCB, males had the largest central complex (Figures 7A and 7B, top row panel). Alate females of *M. brevinoda* did not differ significantly from either males or workers (Figure 7A, top panel and inset). In *R. metallica*, no significant difference was found in the central complex volumes among the two female castes; both were significantly smaller than the alate male (Figure 7B, top panel and inset).

The pattern seen in the central complex of *M. brevinoda*, with males having the largest volumes relative to the RoCB, was also seen in the FB and the noduli (Figure 7A, second and fifth row panel and insets). The EB, however, showed significant differences between all three castes, with the smallest volumes seen in alate females (Figure 7A, third row panel and inset). Lastly, there were no differences in the volume of the protocerebral bridge between the three castes in *M. brevinoda* (Figure 7A, fourth row panel and inset). In *R. metallica*, the FB, EB, protocerebral bridge could not be compared for grade shifts or volumetric differences since the scaling slopes among the three castes were significantly different in each case (Figure 7B, second to fourth row panels and insets). In the noduli, similar to the central complex, we found no significant difference in the volume among the two female castes, both of which were significantly smaller than the alate male (Figure 7B, fifth row; Table 5).

## Discussion

To investigate the relationship between lifestyles, ecologies and brain morphology, we measured neuropil volume across three castes in two species of Australian ants, *M. brevinoda and R. metallica.* We identified variations in neuropil investment patterns throughout the brain, which likely reflect the considerable differences in lifestyle, mode of locomotion, visual ecology, and morphology that each species and caste exhibits. Our results also highlight areas where relative tissue investment appears to be conserved across castes or between species, pointing to shared evolutionary pressures or functional constraints that shape brain organization in these species.

### Sensory processing centers

Our results indicate that investment in the optic lobes varies among different castes, reflecting the unique and varying visual demands faced by alate males, alate females, and workers. We observed significantly larger optic lobe volumes in males when compared to both the female castes in the study species. Male ants of both species also possessed significantly larger lamina, medulla, and lobula compared to their female counterparts. This male-specific increase in investment in visual processing centers has been documented in several other ant species, including *Cataglyphis nodus*,^35^ *Atta vollenweideri*,^36^ and *Camponotus japonicus*.^37^

Males and female alates must identify mating sites, with males relying heavily on their vision to intercept flying females mid-air while also fending off competing males. To achieve mating success while in flight, males require precise, fast, and accurate visual information. Males are known for their reliance on vision, evident from their disproportionately large compound eyes^17^ and ocelli.^18^ Male ants also have a higher number of ommatidia, the individual functional units of a compound eye, compared to females of the same species.^14^^,^^18^ In some species, such as *Camponotus pennsylvanicus* and *Camponotus consobrinus*, both male and female alates have more ommatidia and larger eyes than workers of comparable head size.^19^^,^^38^ A greater number of ommatidia, along with larger lenses and smaller interommatidial angles, increases the spatial resolution of the eye and thus shows the importance of vision in both reproductive castes.^18^^,^^39^^,^^40^^,^^41^^,^^42^^,^^43^

The mode of locomotion may also play a role in defining the investment in the optic lobes. Male alates spend a significant fraction of their lives on the wing, while female alates are also capable of flight. Workers, on the other hand, are strictly pedestrian. Flying may thus necessitate better visual capabilities, such as higher temporal resolution, to allow males and females to locate mates successfully. Other insects that face similar visual processing challenges due to their aerial lifestyles also demonstrate well-developed visual processing centers. For instance, large and complex optic lobes can also be found in dragonflies, which must quickly respond to small, fast-moving targets while hunting.^44^

Our study revealed significant differences in antennal lobe volumes between males and workers of *M. brevinoda*. Males possessed larger antennal lobes, a pattern also observed in other ants, such as *C. nodus*,^35^ *C. japonicus*,^37^ *C. sericeus*, and *C. compressus*.^45^ Males in several insect species, including ants,^36^^,^^37^ honeybees,^46^ hornets,^47^ and moths,^48^^,^^49^ possess enlarged glomeruli (the distinct processing units in the antennal lobe) specialized for detecting sex pheromones. While sex pheromones likely influence the mating behavior of *M. brevinoda*, this remains to be confirmed. Furthermore, no differences were detected in the antennal lobe volumes between the two female castes of *M. brevinoda*. This pattern contrasts with that of honeybees, where queens have slightly smaller antennal lobe volumes compared to workers, resulting in reduced performance in olfactory learning tasks.^50^ Unlike honeybee queens, alate females of *M. brevinoda* engage in foraging and nest maintenance during the establishment phase of their colony,^15^^,^^16^ necessitating a sense of smell comparable to that of workers, who subsequently assume these duties.

In contrast to *M. brevinoda*, there were no significant differences in antennal lobe volume in the three castes of *R. metallica*. Additionally, the antennal lobes in all castes of *R. metallica* were consistently larger relative to RoCB when compared to their counterparts in *M. brevinoda*, suggesting a greater reliance on olfactory cues or perhaps a less complex cuticular hydrocarbon profile.^51^ The larger antennal lobes in *R. metallica* could also be because workers of this species engage in calling behavior to attract males.^31^ Similarly, diurnal and nocturnal dung beetles that strongly rely on olfactory cues for many key behaviors, such as finding specific types of dung to feed on, also do not show differences in antennal lobes, despite differences in behavior.^52^

Generally, a trade-off is noted between the visual and olfactory sensory regions—nocturnal insects invest more heavily in olfactory processing regions, while diurnal insects invest more in visual processing regions (bull ants,^30^ hawk moths,^27^ and paper wasps^28^), thus taking advantage of the readily available cues in their surroundings. Surprisingly, our study reveals an inverse pattern: nocturnal *M. brevinoda* workers had larger visual processing regions than diurnal *R. metallica* workers, while the latter exhibited larger olfactory processing regions.

Larger optic lobes and more facets in the compound eye of the nocturnal *M. brevinoda* workers may be an adaptation to improve vision in dim-light conditions. Workers of *M. brevinoda* have a head width of 3.91 ± 0.11 mm and possess approximately 3,590 ± 88 facets in each compound eye.^53^ Workers of *R. metallica* are significantly smaller, with a head width of 1.44 ± 0.07 mm and an average of 275 ± 12 facets in their eyes.^54^ Thus, relative to their head size, *M. brevinoda* workers have approximately five times more facets than *R. metallica* workers, which suggests they have a significantly higher sampling resolution. Workers of *M. brevinoda* also have larger lenses compared to *R. metallica* (31.62 μm vs. 18.5 μm), which is a visual adaptation to enhance the light gathering capacity of the eye, essential for dim light conditions.^18^^,^^53^^,^^54^

### Higher-order integration

In insect brains, mushroom bodies are centers of multimodal associative learning and play a fundamental role in visual navigation.^55^^,^^56^^,^^57^^,^^58^^,^^59^ In our study, males of both species possessed the smallest mushroom bodies. Since males perish shortly after copulation,^60^ they do not need to acquire visual memories, such as those used to locate a nest after a foraging trip, leading to lower investment in memory centers,^61^ a pattern also seen in termites^62^ and other ants, such as *C. nodus*.^35^

In contrast, workers of both *M. brevinoda* and *R. metallica* possess the largest mushroom bodies among the three castes. Workers perform diverse tasks requiring significant behavioral flexibility, particularly in the domain of associative memory. For example, *M. brevinoda* foragers learn and memorize visual panoramas and landmarks to locate their nest following a foraging trip.^63^ Similarly, young queens of both *R. metallica*^34^ and *M. brevinoda*^15^^,^^16^ also face navigational challenges for a brief period of their lives while establishing new nests. Visual homing requires better processing capabilities in brain areas involved with visual image recognition, which likely contributes to the observed differences in mushroom body volume between castes.

Consistent with this, the mushroom body calyx in ants receives segregated sensory input, with the lip dominated by olfactory projections and the collar by visual projections. In both species, workers and alate females possess significantly larger lip regions, suggesting the two female castes invest most in olfactory associative memory. The underlying reason for the volumetric differences in the calyces between castes may be similar to that in *C. nodus*: workers and queens possess nearly double the number of microglomeruli in the mushroom body calyces compared to males.^35^ While statistical comparisons of collar size between castes were not possible, workers of *M. brevinoda* had a significantly larger relative collar regions (relative to the lip) compared to *R. metallica*, which reflects the strong reliance of vision in *Myrmecia* ants.^15^^,^^56^

The central complex is involved in many functions, including modulating internal states (such as sleep), transforming sensory signals into internal representations of head direction, encoding travel direction, and initiating steering commands (reviewed in the study by Heinze et al.^10^). Across insects, the central complex is highly morphologically conserved.^9^^,^^64^^,^^65^ Males of both species studied here possessed the largest central complexes, which aligns with previous observations in other ant species.^35^^,^^36^^,^^37^ Of the four central complex neuropils, the greatest difference was observed in the noduli. The noduli are innervated by neurons that encode rotational and translational speed, as well as neurons that project to the FB and are involved in encoding travel direction.^66^^,^^67^^,^^68^ Enlarged noduli may be attributable to the fact that males spend most of their active life in flight and therefore rely more on accurately monitoring their flight speed and travel direction. Additionally, male ants need to target fast-flying objects in a cluttered background while competing with other males. This increased investment in the central complex has been suggested to “promote efficacy in male mating behavior” among other advantages.^35^

In summary, we found clear differences in the relative volumes of functionally distinct brain regions both between species and castes. Male ants that have a short lifespan and a specialized task of locating mates invest significantly less in brain regions that support memory and learning but an increased investment in optic lobes. Female castes that have a longer lifespan and exhibit a large behavioral repertoire, invest significantly in the mushroom body, a brain region that supports learning and memory. Between the two species, males and workers of *M. brevinoda* had relatively smaller antennal lobes, compared to *R. metallica*, suggesting that olfaction plays a larger role in the latter. The differential investment in brain regions based on distinct locomotory strategies and lifestyles highlights the need for comparative analyses of both neural and behavioral traits within species.

### Limitations of the study

Our results in this study are based on volumetric analyses of brain regions in three castes of ants that exhibit distinct locomotory strategies. While this allowed us to show similarities and differences in neuropil volumes between castes, it did not account for experience of individuals. It would be important to take this into account in future studies since experience is known to affect size of certain higher order neuropils. Our comparison of all three castes was carried out in two species. The limited taxonomic coverage limits the ability to disentangle broader evolutionary patterns. Sampling across the phylogeny may allow us to identify whether the patterns seen here are conserved in ants. Lastly, we used an accurate but laborious technique involving immunohistochemistry and confocal microscopy, which is not suitable for large scale comparison. Future large scale comparative studies could rely on micro computed tomography techniques for such analyses.

## Resource availability

### Lead contact

Request for further information and resources should be directed to and will be fulfilled by the lead contact, Ajay Narendra (ajay.narendra@mq.edu.au).

### Materials availability

This study did not generate new unique biological materials, cell lines or other reagents.

### Data and code availability

• Data reported in this paper are publicly available at https://tinyurl.com/ypbvbbh7 as of the date of publication.
• Original code used in this paper is publicly available at https://tinyurl.com/ypbvbbh7 as of the date of publication.
• Any additional information required to reanalyze the data reported in this paper is available from the lead contact upon request.

## Acknowledgments

We acknowledge the Traditional Custodians of the land where we carried out our research, the Wallumattagal Clan of the Dharug Nation. We thank Sue Lindsay and Arthur Chien for providing us access to microscopy facilities. The research was supported by an International Research Training Program Scholarship to S.E., and an Australian Research Council Discovery Project grant DP220102836 to A.N.

## Author contributions

Conceptualization, A.N.; methodology, S.E., Z.B.V.S., M.E.S., and A.N.; formal analysis, S.E., M.E.S., and A.N.; investigation, S.E., Z.B.V.S., M.E.S., and A.N. resources, F.P. and A.N. data curation, S.E. and A.N.; writing, original draft, S.E.; writing, review & editing, S.E., M.E.S., F.P., and A.N.; visualization: S.E., M.E.S., and A.N.; funding acquisition: A.N.

## Declaration of interests

The authors declare no competing interest.

## STAR★Methods

### Key resources table

**Table:** 

REAGENT or RESOURCE	SOURCE	IDENTIFIER
**Antibodies**		

3C11 anti SYNORF1	DHSB	RRID: AB_528479
Alexa Fluor 488 goat anti-mouse	ThermoFisher Scientific	RRID: AB_2534062

**Deposited data**		

Confocal scans, labelled data of *Myrmecia brevinoda*	Confocal microscope, AMIRA	https://tinyurl.com/mrv6ft8t
Confocal scans, labelled data of *Rhytidoponera metallica*	Confocal microscope, AMIRA	https://tinyurl.com/cp55kc36
R scripts used in this project	This paper	https://tinyurl.com/mtfazcct

**Experimental models: Organisms/strains**		

*Myrmecia brevinoda*	Wild caught, Sydney, Australia	N/A
*Rhytidoponera metallica*	Wild caught, Sydney, Australia	N/A

**Software and algorithms**		

Amira 6.0.1	https://tinyurl.com/3psdvhzw	N/A
Fiji ImageJ 2.1.0	Schindelin et al.^69^	N/A
Biomedisa	Lösel et al.^70^	https://biomedisa.info
RStudio 2025.05.0+496	RStudio, Inc. Boston, MA, US	https://www.rstudio.com

### Experimental model and study participant details

Omitted as our study does not involve biological models.

### Method details

#### Study species and location

We studied workers, alate males and alate females of *Rhytidoponera metallica* (n = 7, 6, and 8, respectively) and alate males and alate females of *Myrmecia brevinoda* (n = 7 and 6, respectively). Data for *M. brevinoda* workers (n = 28) were obtained from Sheehan et al.^30^ (in this paper *M. brevinoda* was misidentified as *M. midas*). Ants were collected from multiple nests at the Macquarie University Wallumattagal Campus in Sydney, Australia (33°46′10.24″ S, 151°06′39.55″ E) between 2017 and 2023. Workers were collected at the nest entrance or while foraging at different times throughout the year. Alate females and males were collected between late January and mid-April as they left the nest entrance for nuptial flights. Captured animals were immediately dissected, and the samples were processed.

#### Immunohistochemistry and imaging

We processed the samples using the same protocol as described by Sheehan et al.^30^ Animals were anaesthetised on ice before removing the brain from the head capsule. The dorsal surface of the head was photographed with a colour camera (Lumix DMC-FZ1000, Panasonic Australia). From these images, the individual’s head width (HW) was determined by measuring along the widest point of the head.

Brains were dissected from the head capsule in Ringer solution (129 mM NaCl, 6 mM KCl, 4.3 mM MgCl_2_ × 6H_2_O, 5 mM CaCl_2_ × 2H_2_O, 159.8 mM Sucrose, 274 mM D-glucose, 10 mM HEPES buffer, pH 6.7-7). Brains were transferred within 10 minutes to a fixative: 4% paraformaldehyde (PFA) in 0.1 M Phosphate Buffered Solution (PBS). Brains were kept on a shaker in the dark in all the following described washes and incubations. Brains were kept in the fixative for two days at room temperature and washed three times (10 minutes each) in PBS. To assist antibody penetration, specimens were washed with 3% Triton-X in 0.1M PBS (PBST) at room temperature. They were then incubated for one hour with 2% Normal Goat Serum (NGS, Sigma-Aldrich) in PBST. Brains were then incubated in the primary antibody solution, 1:50 anti-synapsin (3C11 anti SYNORF1; DHSB) for four days in 2% NGS in PBST, at room temperature. This was followed by five washes in PBS (10 minutes each). They were then incubated in the secondary antibody solution, 1:250 goat anti-mouse antibody (Alexa Fluor 488 goat anti-mouse, ThermoFisher Scientific), for three days in 1% NGS in PBST, at room temperature. Following this, the brains were washed five times with 0.1 M PBS (10 minutes each) and dehydrated through an ascending series of ethanol (30%, 50%, 70%, 90%, 95% and 100%; 10 minutes each). Brains were cleared for 10 minutes in 1:1 methyl salicylate:ethanol, followed by 100% methyl salicylate (Sigma Aldrich, M6752) for one hour at room temperature. Lastly, brains were transferred to custom-made metal slides containing 1 cm diameter holes. A well was created by sealing the holes with a coverslip on one side. Brains were immersed in 100% methyl salicylate with the ventral side facing upward, and the well was sealed with another coverslip.

Two of the *M. brevinoda* alate female brain samples were collected in 2023 to increase the sample size. These two brains were processed using the above-described protocol, with slight modifications. Following dissection, brains were fixed overnight at 4°C. Additionally, they were incubated in the primary and secondary antibody (1% NGS in PBST) for only two days each. These changes to the original protocol did not appear to have an impact on the fixation or the staining of the tissue. Lastly, to preserve specimens, these brains were mounted in 100% Permount. We did not observe any immediately noticeable differences in synapsin signal between the samples fixed for 48 hours compared to those fixed overnight.

All specimens (except the brains of the two *M. brevinoda* alate females processed in 2023) were imaged using an inverted confocal laser scanning microscope (Olympus FluoView FV 1000 IX81) using a 10x objective (UPlanApo, NA 0.4) and 1-3.1 μm optical sections. The two specimens processed in 2023 were imaged using an Olympus FluoView FV 3000RS IX83 confocal microscope with a 10x objective (UPLSAPO10X2). For ants with large brain size (seen in *M. brevinoda*), we imaged three overlapping z-stacks that were merged together using the “Pairwise Stitching” plugin in the Fiji program^69^ to produce a single image.

### Quantification and statistical analysis

#### Brain volumes

Three-dimensional reconstructions of neuropils were done based on the anti-synapsin labelling to obtain volumes of individual regions. Neuropil boundaries were delineated in every third image in each image stack using Amira (v. 6.0.1, ThermoFisher Scientific). Two methods were used to produce complete representations of these regions: the interpolate function in Amira and a semi-automated interpolation method using ‘Biomedisa’.^70^ These interpolated contours were double-checked and manually corrected in cases where deviations from the neuropil boundaries were noted. The spherical aberration that is known to occur due to refractive index (RI) mismatch between air (RI = 1) and the mounting media was corrected by multiplying the optical section thickness by 1.55 and 1.54 when samples were mounted in methyl salicylate (RI = 1.536) and permount (RI = 1.520), respectively (calculated from Diel et al.^71^). The volume of the outlined regions was calculated using the “MaterialStatistics” function in Amira. Finally, 3D reconstructions were made using a polygonal surface model in Amira.

Four functionally defined areas with clear glial borders were quantified in each brain. These included the optic lobes (OL; containing the Lamina (LA), Medulla (ME) and Lobula (LO)), the Antennal lobe (AL), the Mushroom Body (MB; containing the Calyx Lip (LIP), Calyx Collar (COL) and the Peduncle (PED)) and the Central Complex (CX; containing the fan-shaped body (FB; also referred to as the upper division of the Central Body CBU), the ellipsoid body (EB; also referred to as the lower division of the Central Body CBL), the protocerebral bridge (PB) and the paired Noduli (NO); Figure 1). In our image data, the basal ring could not be reliably distinguished from the COL. Thus, ‘COL’ in this paper refers to the combined volume of the COL and the basal ring. The rest of the brain is composed of undefined neuropil mass and was thus segmented as one single region: the rest of the central brain (RoCB; which includes the unstructured lateral protocerebrum and the subesophageal ganglion). Paired neuropils were traced in only one hemisphere. This volume was multiplied by two to estimate the total volume of bilateral neuropils. The total neuropil volume of the brain was obtained by summing the volume of the paired and unpaired neuropils. No cell bodies were included in the measurements.

#### Data for *M. brevinoda* workers

For workers of *M. brevinoda*, we used the raw image data and label files of the brains (n = 28) from Sheehan et al.^30^ Neuropil volumes were calculated as described earlier to ensure comparability with data collected as part of this study. The spherical aberration of the obtained data was corrected by multiplying the raw optical section thickness by 1.55, as samples in Sheehan et al.^30^ were also mounted in an identical methyl salicylate medium (Sigma Aldrich, M6752; RI = 1.536).

The two axes of comparison in this study were the two species and the castes within these species. To investigate whether differences in neuropil volume between any groups were attributable to genuine differences in neuropil sizes among distinct groups rather than variations caused by allometric scaling, we performed a standardised major axis regression (SMA) analysis on individual neuropils in relation to the unstructured brain volume, the RoCB, using the SMATR^72^ package in R (version 4.3.0). We chose RoCB, as a baseline since it is independent of all the different neuropils which enables identification of the true volumetric differences of neuropils between castes and species. This analysis assumes an allometric relationship described as y = α · x to the power b, which can be translated to the linear relationship log(y) = log(x)·b+log(α). If the allometric scaling or the slopes between the tested groups were not statistically different, we tested for differences in the y-axis intercept or elevation, which implies a true difference in neuropil volumes across groups. Thus, all comparisons between groups using SMA refer to differences in neuropil volume relative to the reference neuropil volume, i.e., the RoCB. Lastly, a grade shift index (gsi), described as α1/α2^12^, was calculated to estimate the magnitude of the shift in elevation, where ‘1’ and ‘2’ represent the two groups being compared. In addition, since the collar and lip of the mushroom body are functionally connected to the optic lobes and antennal lobes, respectively, we performed SMA analysis following the same procedure between the collar and optic lobes, lip and antennal lobes and collar and lip, both between and within both species (Tables S2 and S3).

## Supplemental information

Document S1. Figures S1 and S2 and Tables S2 and S3

Table S1. Absolute volume of neuropils and head width of workers, alate males (males) and alate females (females) of *Myrmecia brevinoda* and *Rhytidoponera metallica*
