# Supplementary material for: Caste- and sex-specific differential investment in brain regions of Australian ants
Source: iScience. 2026 May 23;29(6):116039. doi: 10.1016/j.isci.2026.116039 (PMC13224040; doi:10.1016/j.isci.2026.116039)
Supplement: Document S1. Figures S1 and S2 and Tables S2 and S3 [file mmc1.pdf]

iScience, Volume *29*

Supplemental information

Caste- and sex-specific differential investment in brain regions of Australian ants

Saroja Ellendula, Zachary B.V. Sheehan, Marcel E. Sayre, Fleur Ponton, and Ajay Narendra

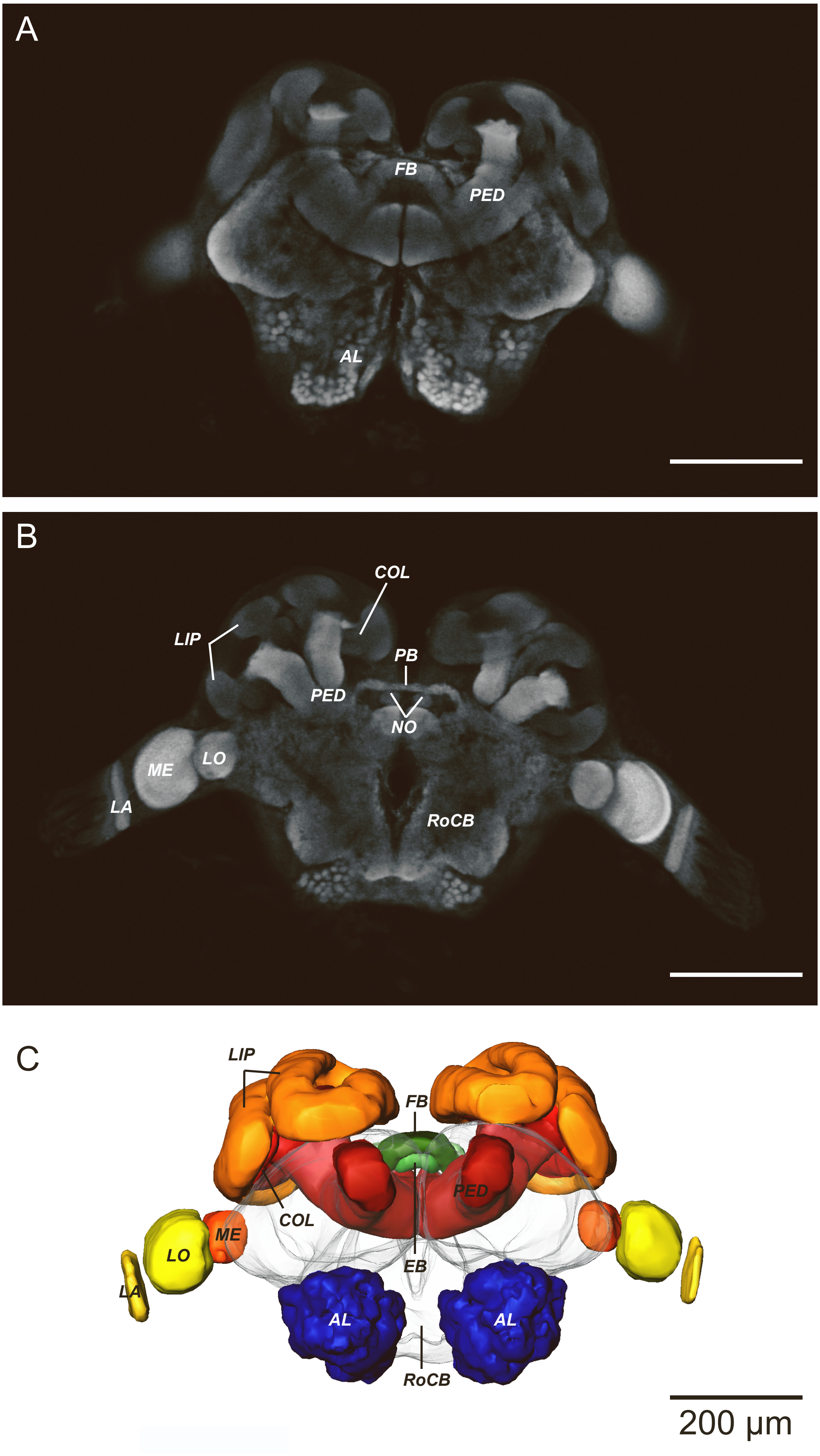

Figure S1: General layout of the ant brain, alate female of *Rhytidoponera metallica*. (A-B) Frontal section of the brain labelled with anti-synapsin highlighting the major neuropils included in this study: optic lobe subregions (lamina [LA], medulla [ME], lobula [LO]), antennal lobe (AL), mushroom body subregions (lip [LIP], collar [COL], peduncle [PED]), central complex subregions (fan-shaped body [FB], ellipsoid body [EB], protocerebral bridge [PB], noduli [NO]), and the rest of the central brain (RoCB). (A) Anterior view, (B) posterior view and (C) reconstruction of the same brain are shown. Scale bars = 200 µm.

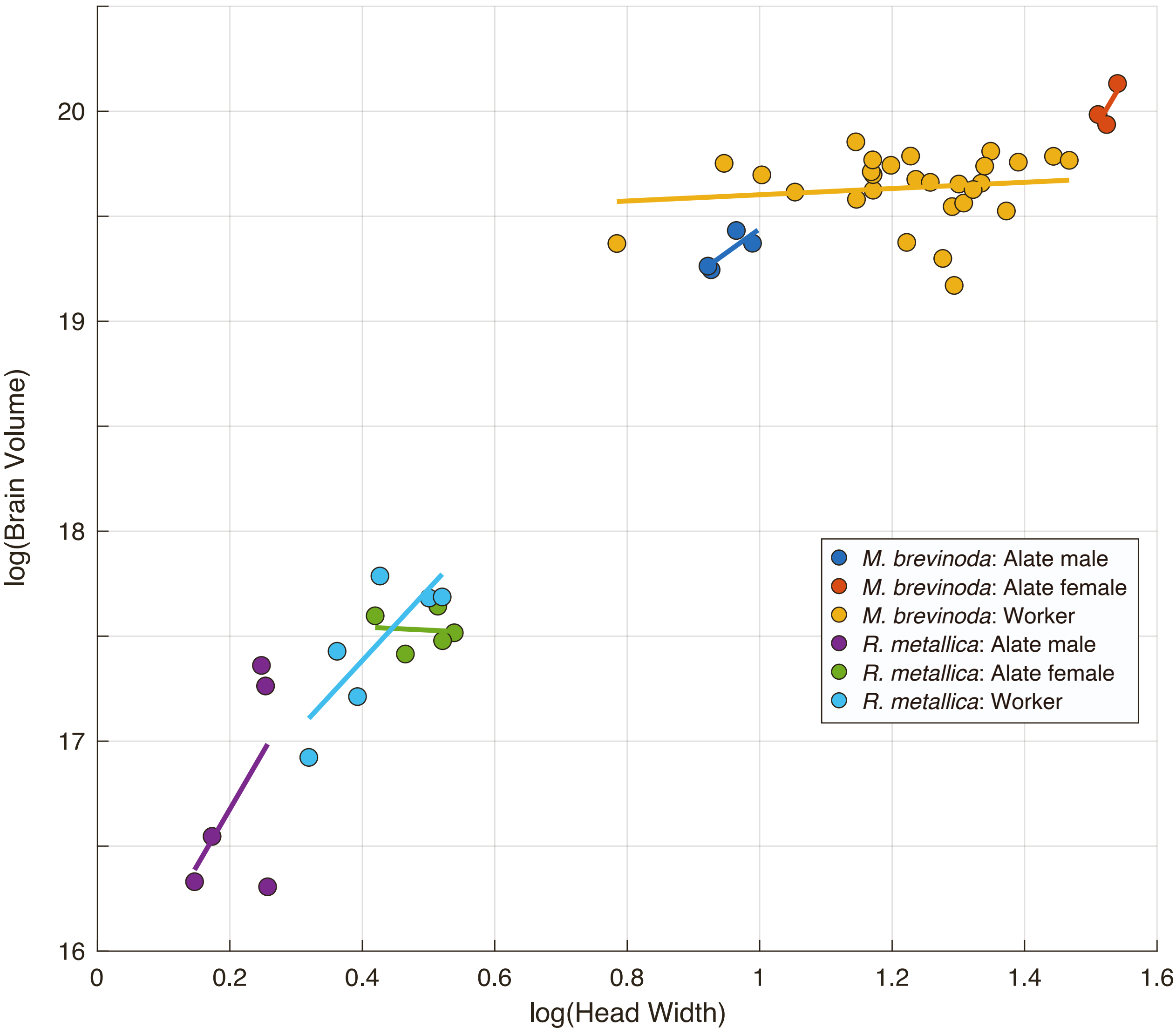

Figure S2: Relationship between head width and total brain volume in three castes of two ant species. Data are log-transformed. Each circle represents data from one individual.

Table S2. Scaling relationship of the lip and the collar against the optic lobe, antennal lobe or each other, between two ant species. Outputs of standardized major axis regression (SMA) on log transformed data (log y = α + βlog x) comparing the scaling relationship of the lip and the collar against the optic lobe, antennal lobe or each other, between the two species, *Myrmecia brevinoda* and *Rhytidoponera metallica*. Abbreviations and gsi conventions are described in STAR Methods.

**Caste**	**y**	**Reference structure**	**Do groups have a common slope? H0 = slopes are equal**	**Is the common slope different from 1 H0 = common slope not different from one**	**Is the lm for *R. metallica* significant?**	**Is the lm for *M. brevinoda* significant?**	**Are there differences in elevation? H0: no difference in elevation**	$\alpha_{R. metallica}$	$\alpha_{M. brevinoda}$	**gsi *M. bevinoda/ R. metallica***
Male	COL	OL	Yes p = 0.432	No p = 0.163	Yes p = 0.014	No p = 0.152	Yes Wald stat = 3.99 $p = 4.56 \times 10^{-2}$	-2.82	-3.14	0.48
Female	COL	OL	Yes p = 0.382	Yes p = 0.004	No p = 0.380	Yes p < 0.01	Yes Wald stat = 15.92 $p = 6.599 \times 10^{-5}$	-5.32	-6.41	0.08
Worker	COL	OL	Yes p = 0.386	No p = 0.153	No p = 0.130	Yes p < 0.01	No Wald stat = 2.509 p = 0.114	0.73	0.97	1.75
Male	LIP	AL	Yes p = 0.120	No p = 0.111	Yes p < 0.01	No p = 0.076	Yes Wald stat = 17.45 $p = 2.96 \times 10^{-5}$	1.39	1.78	2.46
Female	LIP	AL	Yes p = 0.886	No p = 0.332	No p = 0.178	Yes p < 0.01	No Wald stat = 0.3338 p = 0.563	-1.24	-1.3	0.88
Worker	LIP	AL	Yes p = 0.469	No p = 0.368	Yes p < 0.01	Yes p < 0.01	Yes Wald stat: 8.769 p = 0.003	0.92	1.08	1.45
Male	COL	LIP	Yes p = 0.789	Yes p = 0.023074	Yes p < 0.01	No p = 0.922	Yes Wald stat = 11.62 $p = 6.52 \times 10^{-4}$	-1.27	-1.5	0.589
Female	COL	LIP	Yes p = 0.201	Yes p = 0.063608	No p =0.289	Yes p < 0.01	Yes Wald stat = 10.1 $p = 1.48 \times 10^{-3}$	-3.44	-2.98	2.91
Worker	COL	LIP	Yes p = 0.553	No p = 0.22916	No p = 0.571	Yes p < 0.01	Yes Wald stat = 106.8 $p < 2.22 \times 10^{-16}$	-0.24	0.52	5.83

Table S3. Scaling relationship of the lip and the collar against the optic lobe, antennal lobe or each other within the castes of two ant species. Outputs of standardized major axis regression (SMA) on log transformed data (log y = α + βlog x) comparing the scaling relationship of the lip and the collar against the optic lobe, antennal lobe or each other, within the castes of *Myrmecia brevinoda* and *Rhytidoponera metallica*. Abbreviations and gsi conventions are described in STAR Methods.

Species	y	Reference structure	Do groups have a common slope? H0 = slopes are equal	Pair wise difference in slope caste1, caste 2, p value			Is the common slope different from 1 H0 = common slope not different from one	Is the linear model significant? (p-values) Yes, p < 0.05 No, p > 0.05	Are there differences in elevation? H0 : no difference in elevation	Pair wise difference in elevation caste1, caste 2, p value			$\alpha_{Workers}$	α_{Female}	α_{males}	gsi (w/f)	gsi (f/m)	gsi (m/w)
M. brevinoda	COL	OL	No p = 0.0017	Male	Female	9.98×10^{-1}		Males = 0.15 Workers < 0.01 Female < 0.01										
				Male	Worker	7.07×10^{-2}												
				Female	Worker	1.83×10^{-3}												
	LIP	AL	Yes p = 0.0939				No p = 0.2130	Males = 0.08 Workers < 0.01 Female < 0.01	Yes Wald stat = 267.9 $p < 2.22 \times 10^{-16}$	Male	Female	$< 2 \times 10^{-16}$	0.17	0.08	-0.24	1.22	2.1	0.39
										Male	Worker	$< 2 \times 10^{-16}$						
										Female	Worker	6.95×10^{-3}						
	COL	LIP	Yes p = 0.1289				No p = 0.2079	Males = 0.93 Workers < 0.01 Female < 0.01	No Wald stat: 1.092 p = 0.58				-0.2	-0.3	-0.19	1.1	0.87	1.04
R. metallica	COL	OL	Yes p = 0.263				No p = 0.1051	Males = 0.01 Workers = 0.13 Female = 0.38	Yes Wald stat = 62.56 $p = 2.609 \times 10^{-14}$	Male	Female	2.36×10^{-7}	-2.2	-2.4	-2.79	1.73	2.51	0.23
										Male	Worker	1.32×10^{-14}						
										Female	Worker	7.68×10^{-4}						
	LIP	AL	Yes p = 0.2153				No p = 0.251	Males < 0.01 Workers < 0.01 Female = 0.18	Yes Wald stat = 128.6 $p < 2.22 \times 10^{-16}$	Male	Female	$< 2.22 \times 10^{-16}$	1.19	1.12	0.69	1.17	2.74	0.31
										Male	Worker	$< 2.22 \times 10^{-16}$						
										Female	Worker	4.16×10^{-3}						
	COL	LIP	Yes p = 0.1509				Yes p = 0.0095	Males < 0.01 Workers = 0.57 Female = 0.29	Yes Wald stat = 330.5 $p < 2.22 \times 10^{-16}$	Male	Female	$< 2.22 \times 10^{-16}$	-2.2	-2.2	-1.27	1.21	0.11	7.49
										Male	Worker	$< 2.22 \times 10^{-16}$						
										Female	Worker	0.39						
